# Prevalence and Impact of Long-term Use of Nicotine Replacement Therapy in UK Stop-Smoking Services: Findings From the ELONS Study

**DOI:** 10.1093/ntr/ntw258

**Published:** 2016-09-24

**Authors:** Lion Shahab, Fiona Dobbie, Rosemary Hiscock, Ann McNeill, Linda Bauld

**Affiliations:** 1 Department of Epidemiology and Public Health, University College London and UK Centre for Tobacco and Alcohol Studies, London, UK; 2 School of Health Sciences, University of Stirling and UK Centre for Tobacco and Alcohol Studies, Stirling, UK; 3 School for Health, University of Bath and UK Centre for Tobacco and Alcohol Studies, Bath, UK; 4 Addictions Department, Institute of Psychiatry, Psychology and Neuroscience, King’s College London and UK Centre for Tobacco and Alcohol Studies, London, UK

## Abstract

**Background:**

Nicotine replacement therapy (NRT) was licensed for harm reduction in the United Kingdom in 2005, and guidance to UK Stop-Smoking Services (SSS) to include long-term partial or complete substitution of cigarettes with NRT was issued in 2013. Yet, NRT prevalence data and data on changes in biomarkers associated with long-term NRT use among SSS clients are scarce.

**Methods:**

SSS clients abstinent 4 weeks postquit date were followed up at 12 months. At baseline standard sociodemographic, smoking and SSS use characteristics were collected and of those eligible, 60.6% (1047/1728) provided data on smoking status and NRT use at follow-up. A subsample also provided saliva samples at baseline and of those eligible, 36.2% (258/712) provided follow-up samples. Saliva was analyzed for cotinine (a metabolite of nicotine) and alpha-amylase (a stress biomarker).

**Results:**

Among those who had used NRT during their initial quit attempt (61.5%, 95% CI 58.4%–64.6%), 6.0% (95% CI 4.3%–8.3%) were still using NRT at 1 year, significantly more ex-smokers than relapsed smokers (9.5% vs. 3.7%; *p* = .005). In adjusted analysis, NRT use interacted with smoking status to determine change in cotinine, but not alpha-amylase, levels (Wald χ^2^ (1) = 13.0, *p* < .001): cotinine levels remained unchanged in relapsed smokers and ex-smokers with long-term NRT use but decreased in ex-smokers without long-term NRT use.

**Conclusions:**

Long-term NRT use is uncommon in SSS clients, particularly among relapsed smokers. Its use is associated with continued high intake of nicotine among ex-smokers but does not increase nicotine intake in smokers. It does not appear to affect stress response.

**Implications:**

Little is known about the long-term effects of NRT. Given an increasing shift towards harm reduction in tobacco control, reducing the harm from combustible products by partial or complete substitution with noncombustible products, more data on long-term use are needed. This study shows that in the context of SSS, clients rarely use products for up to a year and that NRT use does not affect users’ stress response. Ex-smokers using NRT long-term can completely replace nicotine from cigarettes with nicotine from NRT; long-term NRT use by continuing smokers does not increase nicotine intake. Long-term NRT appears to be a safe and effective way to reduce exposure to combustible nicotine.

## Introduction

The main aim of the UK Stop-Smoking Services (SSS) is to support attempts to quit smoking. However, not all smokers either feel able to or want to stop smoking completely. For this reason, alternative approaches have been explored to reduce harm from smoking in this population. Harm reduction refers to the reduced psychological or physiological harm from substance use without complete cessation.^1^ For current smokers, harm reduction may refer to the partial substitution of cigarettes with noncombustible forms of nicotine delivery such as nicotine replacement therapy (NRT) to reduce cigarette consumption or for temporary abstinence. For ex-smokers, harm reduction constitutes the complete, long-term substitution of combustible tobacco products (eg, cigarettes) with less harmful noncombustible nicotine delivery devices.^2^ There is good evidence from both population studies and clinical trials that the provision of NRT to smokers who cut down their cigarette consumption results in more sustained decreases in cigarette consumption and improves their chances to stop smoking completely.^3,4^ It increases motivation to stop and improves quit rates^1,3^ but does not increase overall nicotine intake.^5,6^ Trials have also shown that extended use of NRT by ex-smokers may result in better long-term abstinence rates by reducing relapse.^7,8^ For these reasons, NRT has been licensed for harm reduction in the United Kingdom since 2005.^9,10^ Based on a previous report,^11^ guidance was also issued in 2013 requiring SSS to include partial or complete long-term substitution of cigarettes with NRT in tailored quit plans for smokers who have difficulty stopping smoking completely so as to help them reduce consumption with the eventual aim to stop smoking.^12^

The vast majority of the harm from smoking is caused by the burning of tobacco and not nicotine.^13^ Thus NRT as a substitute for cigarettes is important to study. Although the importance of e-cigarettes for harm reduction purposes cannot be doubted, NRT is likely to remain a major component of harm-reduction strategies, given its long history in tobacco control and continuing NRT product innovation^14^ and on-going resistance of some smokers to e-cigarettes.^15^ Despite being an established treatment, there is considerable worry among potential users^16^ and stop-smoking advisors^17^ regarding the safety of long-term NRT use, possibly due to misunderstandings about the role of nicotine separate from smoked tobacco.^18^ While studies which have looked at this issue find that long-term NRT use is safe and any associated health risks small,^19^ certainly compared with continued smoking,^20,21^ most data come from clinical trials, which have samples that tend to differ in important ways from general population samples, biasing outcomes.^22^ Given recent calls for further research in the area of harm reduction,^12^ more studies on real-world use are required.

A recent population-based study suggested that only a small percentage of ex-smokers continue to use NRT beyond the standard length of 3 months and that long-term use is associated with lower nicotine intake compared with smokers.^6^ However, in many industrialized countries most NRT is purchased over the counter,^23^ rather than coupled with specialist behavioral support, which is more effective.^24^ Therefore, existing findings may not generalize to smokers attending SSS, especially since in this context the NRT provided is either free or heavily subsidized. In light of the recent broadening in the provision of NRT in SSS, there remains a need to evaluate harm reduction with NRT in this context.

This study describes the impact of longer-term NRT use among smokers who made a quit attempt with SSS support and agreed to take part in the “*E*valuating *L*ong Term *O*utcomes of *N*HS *S*top-Smoking Services” (ELONS) study conducted 2012–2014.^25^ Participants were followed up for 1 year and provided information on their NRT use. A subset also provided saliva samples which were analyzed for two biomarkers of interest: cotinine, the primary metabolite of nicotine as a biomarker of exposure; and alpha-amylase, a digestive enzyme and indicator of autonomic nervous system activation which correlates with acute and chronic stress, as a biomarker of risk/potential harm.^26^ We included this biomarker as animal research has shown that chronic nicotine self-administration can increase stress response in rodents.^27,28^ Specifically, this study aimed to answer the following research questions:

1. What is the prevalence of long-term NRT use among smokers and ex-smokers who had attempted to stop smoking using SSS?
2. What is the impact of long-term NRT use on biomarkers of nicotine exposure and stress among smokers and ex-smokers who had attempted to stop smoking using SSS?

## Methods

### Study Design and Participants

Given the aims of this study, we report only on those with baseline and follow-up data. Full details of the study design and sampling are provided elsewhere.^25^ Briefly, as part of the ELONS study, clients participating in English SSS who set a quit date were asked if they were interested in taking part in a long-term (12 months) evaluation of the services by advisors and informed consent was obtained from all participants, resulting in a baseline sample of 3045 clients. As per standard NHS SSS guidelines, smoking status was recorded at 4-week follow-up^29^ and only those who were abstinent at 4 weeks (56.7%; 1728/3045) were eligible for long-term follow-up. Of all eligible participants for 12 month follow-up, 60.6% (1047/1728) could be contacted by telephone to assess smoking status and NRT use, thus providing complete baseline and follow-up questionnaire data (see Table 1 for participant details). Of those contactable, 53.3% (558/1047) self-reported as abstinent and were eligible for a home visit to verify their smoking status, of whom 4.6% (26/558) failed carbon monoxide verification and were therefore reclassified as smokers for the purposes of this analysis. The 12-month follow-up started in April 2013 and finished in March 2014.

**Table 1.** Baseline Characteristics

	Questionnaire data		Biomarker data	
	Available (*N* = 1047)	Lost to follow-up (*N* = 681)	Available (*N* = 258)	Lost to follow-up (*N* = 454)
Sociodemographic/health characteristics				
Mean (*SD*) Age	46.4 (14.0)	41.1 (13.7)***	45.7 (13.4)	42.2 (14.6)**
% (*N*) Female	55.0 (576)	53.6 (365)	51.6 (133)	48.7 (221)
% (*N*) White	97.2 (1018)	94.7 (645)**	96.5 (249)	93.8 (426)
% (*N*) Cohabiting	53.4 (559)	47.3 (322)*	53.9 (139)	44.9 (204)*
% (*N*) Routine/manual occupation	30.9 (323)	34.5 (235)	25.2 (65)	30.6 (139)
% (*N*) Degree or equivalent	10.6 (111)	10.4 (71)	10.5 (27)	9.3 (42)
% (*N*) Medical condition	59.5 (622)	52.9 (360)**	57.4 (148)	58.4 (265)
Smoking characteristics				
Mean (*SD*) Heaviness of smoking index	3.28(1.45)	3.22 (1.46)	3.19 (1.54)	3.51 (1.41)**
% (*N*) Smoking length < 10 years	10.9 (114)	17.4 (118)***	9.3 (24)	14.3 (65)
% (*N*) Quit attempt last 12 months	41.7 (434)	41.0 (275)	38.1 (98)	38.4 (172)
NHS SSS treatment characteristics				
% (*N*) Intervention type*				
Closed group	3.2 (34)	2.9 (20)	6.6 (17)	4.6 (21)
Open (rolling) group	20.8 (218)	17.6 (120)	21.3 (55)	13.9 (63)
Drop-in clinic	26.5 (277)	27.2 (185)	24.4 (63)	30.4 (138)
One to one support	49.2 (515)	51.9 (353)	47.7 (123)	50.7 (230)
Other	0.3 (3)	0.3 (2)	0 (0)	0.4 (2)
% (*N*) Medication**				
Single NRT	17.4 (182)	17.9 (122)	17.4 (45)	15.2 (69)
Combination NRT	12.2 (128)	15.1 (103)	16.3 (42)	27.3 (124)
Varenicline	50.2 (526)	48.5 (330)	48.4 (125)	37.4 (170)
Other^a^	19.0 (199)	17.0 (116)	16.3 (42)	19.2 (87)
None	1.1 (12)	1.5 (10)	1.6 (4)	0.9 (4)

NRT = nicotine replacement therapy; SSS = UK Stop-Smoking Services.

^a^Bupropion and mixed medication (mainly NRT).

*p < .05; **p < .01; ***p < .001.

A subsample of participants also provided a saliva sample at baseline, before their target quit date (61.6%; 1875/3045). Of those who were eligible to provide a saliva sample at follow-up (ie, successful quitters at 4 weeks with a baseline saliva sample who self-reported abstinence at 12-month follow-up and therefore had a home visit), 52.8% (169/320) provided a sample. Because relapsers did not have a home visit (and therefore were not asked to provide a saliva sample), an additional random selection of participants with baseline saliva samples who had relapsed at 4-week follow-up were contacted at 12 months (83.4%, 392/470) to obtain follow-up saliva samples from smokers. Participants were sent a saliva kit through the post and asked to return samples directly to UCL. The saliva kit contained two Sarstedt Salivettes, a letter from the Principal Investigator asking for their help, detailed instructions on sample collection and a £10 shopping voucher. Of those approached, 22.6% (89/392) returned a saliva sample, resulting in an overall response rate from face-to-face or postal collection of 36.2% (258/712) with complete baseline and follow-up biomarker data (see Table 1 for participant details).

### Measures

#### Questionnaire Items

In addition to standard questions on smoking and sociodemographic characteristics, a number of items were included in the baseline questionnaire to help evaluate SSS.^25^ Advisors recorded the types of pharmacotherapy and behavioral intervention used during the quit attempt. It should be noted that at the time of the study, e-cigarettes (another harm reduction tool) were only just becoming popular and client use was not routinely recorded by SSS. At 12-month follow-up, questions related to long-term NRT use were also assessed retrospectively: participants were asked to indicate whether they had used NRT for their initial quit attempt and, if so, how long they had used NRT for, and if they were still using NRT now. As the use of other nicotine-containing products (including e-cigarettes) was not assessed at baseline, this was assessed at follow-up only. In order to ascertain smoking status and use of NRT in those participants who provided a saliva sample through the post and did not receive a home visit, these respondents were asked to indicate on a tick box included on the salivettes whether they were currently smoking (yes/no) and used NRT or e-cigarettes (yes/no).

#### Biomarkers

Saliva samples were collected with Sarstedt Salivettes and stored in −20°C freezers, ready for analysis. Saliva was analyzed for cotinine by ABS laboratory using rapid liquid–gas chromatograpy^30^ and for alpha-amylase activity by Salimetrics laboratory using an established enzyme-kinetic methodology.^31^ Although alpha-amylase activity is largely independent of flow-rate,^32^ all participants were instructed to keep the salivettes in the mouth for the same amount of time (1–2 minutes) without chewing as per recommendation.^33^ In addition, all participants were asked to abstain from drinking or eating immediately before providing a sample. Whilst alpha-amylase exhibits a diurnal pattern, it remains relatively stable throughout the day following a rise in the first hours after waking.^34^ Participants were therefore instructed to provide two samples during waking hours, approximately 10 minutes apart to increase reliability of measurement (the average coefficient of variation in alpha-amylase activity at baseline was 1.7% and at follow-up 1.8%).

### Analysis

Data were analyzed with IBM SPSS Statistics 20.0.0. Comparisons were made between those who did and did not have complete baseline and follow-up data for questionnaire items (to assess NRT prevalence) and those who did or did not have complete baseline and follow-up biomarker data (to assess impact). Differences were assessed with chi-square tests and independent *t* tests for categorical and continuous variables, respectively. In the prevalence analysis, descriptive statistics including 95% confidence intervals (95% CI) were calculated and, where applicable, groups compared using logistic regression. To correct for nonresponse all prevalence estimates are weighted.^25^

In the biomarker analysis, due to the typically positively skewed distribution of cotinine and alpha-amylase values and relatively small sample size, geometric means and interquartile ranges were calculated. The nonparametric Kruskal-Wallis and Wilcoxon tests were used to assess between-group differences and within-group differences (to look at change across time), respectively. In sensitivity analysis, findings were re-examined with generalized linear models for between- and within-group comparisons that used a gamma distribution with a log link (all zero values were replaced with 0.001) to account for the non-normal distribution and adjusted for potential confounders (age, sex, ethnicity, occupation, any medical condition and nicotine dependence). Statistical significance was set at the standard level (*p* < .05), and the Bonferroni correction was applied to account for multiple comparisons and Type I error rate. The study received ethical approval from the South East Scotland Research Ethics Committee (11/AL/0256) and was carried out in accordance with the ethical principles on human research, as set out in the Declaration of Helsinki.

## Results

### Prevalence of Long-term NRT Use Among Current Smokers and Ex-smokers

Information on long-term NRT use was provided by 1047 participants (34.4% of the total ELONS sample) who constitute the analytic sample for the prevalence analysis. Those who were lost at follow-up were younger, had smoked for a shorter period, were less likely to have a medical condition, to be white or cohabiting (Table 1). All prevalence estimates in this section are weighted.

**Figure 1.**
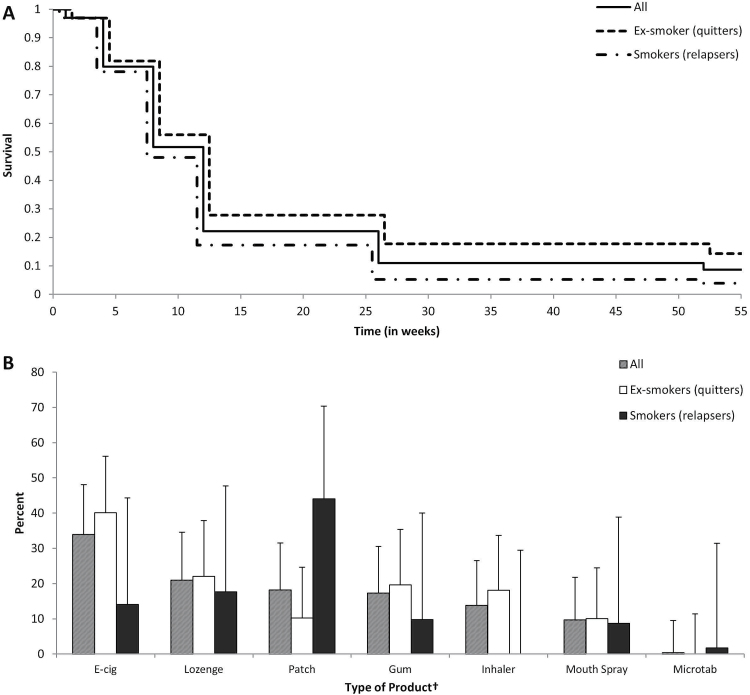
(A) Nicotine replacement therapy (NRT) use across follow-up period among those who had used NRT during initial quit attempt (*N* = 583)*; (B) Product type used among those with long-term NRT use at follow-up (*N* = 50). *Includes e-cigarettes (users of products at 12-month provide denominator for Figure 1B); †No use of nicotine nasal spray reported at 12-month follow-up; users could indicate multiple products; Error bars are 95% confidence intervals.

Of clients followed-up, 61.5% (95% CI 58.4%–64.6%, *N* = 583) reported using NRT during their initial quit attempt. This figure was somewhat higher than the recorded NRT use in SSS (around *N* = 500 when including the “Other” category in Table 1), suggesting that some participants had obtained additional NRT over the counter. Figure 1A provides a breakdown of clients in terms of the length of use of NRT and as a function of smoking status at follow-up. As can be seen, most clients who started on NRT used it for at least 8 weeks and more than one in five (21.5%, 95% CI 18.3%–25.0%, *N* = 137) for longer than the standard 12 weeks. However, long-term use was relatively rare with less than 1 in 10 participants still using noncombustible nicotine delivery devices at 12-month follow-up (8.4%, 95% CI 6.4%–11.0%, *N* = 50), including both NRT and e-cigarettes. In this sample, NRT use was twice as prevalent (6.0%, 95% CI 4.3%–8.3%, *N* = 35) as use of e-cigarettes at 12 months (2.9%, 95% CI 1.8%–4.7%, *N* = 18; some participants were dual product users).

Generally, the pattern of NRT use across the study period was relatively similar for those who had remained abstinent and those who had relapsed by 12-month follow-up (Figure 1A). However, ex-smokers had higher rates of NRT use compared with relapsers at all time-points. At 12-month follow-up, long-term ex-smokers were over four-times more likely than relapsers to be still using noncombustible nicotine delivery devices (*OR* 4.25, 95% CI 2.15–8.40, *p* < .001): 14.0% (95% CI 10.3%–18.7%, *N* = 38) of ex-smokers were still using these compared with 3.7% (95% CI 2.0%–6.5%, *N* = 12) of relapsers. This difference, while being attenuated, remained significant when excluding those who used e-cigarettes only (*OR* 2.91, 95% CI 1.38–6.11, *p* = .005) with 9.5% (95% CI 6.4%–13.8%, *N* = 25) of ex-smokers and 3.5% (95% CI 1.9%–6.3%, *N* = 10) of relapsers still using NRT, respectively. Comparing the quitters and relapsers who were or were not using NRT at follow-up in terms of the characteristics presented in Table 1 showed that dependence was the only variable (other than medication use, as would be expected) that differed between groups (*F*(3, 1037) = 5.52, *p* < .001). Relapsers without NRT use had significantly higher dependence scores than quitters, irrespective of their NRT use.

When looking at individual nicotine-delivery devices still used at 12-month follow-up, e-cigarettes were the most popular, followed by the nicotine lozenge, patch, and gum (Figure 1B). No one used the nasal spray, possibly due to the higher cost of the nasal spray compared with other NRT products, and 16.8% were using multiple products. Due to the small numbers involved, there was insufficient power to detect meaningful differences between those who had remained abstinent and those who had relapsed.

### Impact of Long-term NRT Use on Biomarkers of Nicotine Exposure and Stress Among Current Smokers and Ex-smokers

Baseline and follow-up saliva samples were provided by 258 participants (8.5% of the total sample) who constitute the analytic sample for the biomarker analysis. Those lost to follow-up were younger, less likely to be cohabiting and there were some differences in the treatments used; they were also more dependent (Table 1).

There were no differences in baseline cotinine levels between any of the groups (Table 2). This was confirmed in adjusted analysis controlling for potential confounders which showed that older age (Wald χ^2^ (1) = 6.6, *p* = .011) and greater dependence (Wald χ^2^ (1) = 26.7, *p* < .001) were the only significant predictors of baseline cotinine levels. Similarly, there were no group differences in baseline alpha-amylase levels, again confirmed in adjusted analysis (Table 2). This showed that older age (Wald χ^2^ (1) = 10.6, *p* = .001), being non-white (Wald χ^2^ (1) = 5.3, *p* = .022) and having any medical condition (Wald χ^2^ (1) = 9.8, *p* = .002) were associated with higher alpha-amylase activity at baseline.

**Table 2.** Biomarker Results by Follow-up NRT Use and Follow-up Smoking Status

	Smokers (relapsers)		Ex-smokers (quitters)	
	NRT use (*N* = 18)	No NRT use (*N* = 73)	NRT use (*N* = 14)	No NRT use (*N* = 153)
Baseline assessment				
Geometric mean (IQR/*n*) cotinine in ng/mL	193.7 (323.1/*17*)	241.1 (238.8/*68*)	340.1 (163.9/*13*)	197.6 (174.6/*146*)
Geometric mean (IQR/*n*) alpha-amylase in U/mL	20.1 (59.1/*12*)	21.8 (27.2/*45*)	29.1 (14.2/*11*)	23.6 (30.5/*109*)
Follow-up assessment				
Geometric mean (IQR/*n*) cotinine in ng/ml	210.8 (240.0/*16*)^a^	244.7 (198.7/*69*)^a^	169.9 (449.6/*10*)^a^	1.2 (21.6/*149*)^b^
Geometric mean (IQR/*n*) alpha-amylase in U/mL	25.8 (69.6/*13*)	26.7 (32.2/*43*)	22.4 (49.7/*10*)	27.6 (37.7/*111*)

IQR = interquartile range; NRT = nicotine replacement therapy.

^a,b^Different letters indicate significant differences between groups(*p* < .05).

At follow-up, there was a clear difference between groups in cotinine levels (Kruskal Wallis *H* (3) = 130.2, *p* < .001). Ex-smokers using no NRT had significantly lower cotinine values at follow-up than all other groups (Table 2). Adjusted analysis confirmed these group differences (Wald χ^2^ (3) = 78.9, *p* < .001) and showed baseline nicotine dependence as the only additional significant predictor of follow-up cotinine levels (Wald χ^2^ (1) = 15.4, *p* < .001). There were no group differences in follow-up alpha-amylase levels which was confirmed in adjusted analysis (Table 2); only greater nicotine dependence at baseline was positively associated with follow-up alpha-amylase activity (Wald χ^2^ (1) = 8.1, *p* = .004).

**Figure 2.**
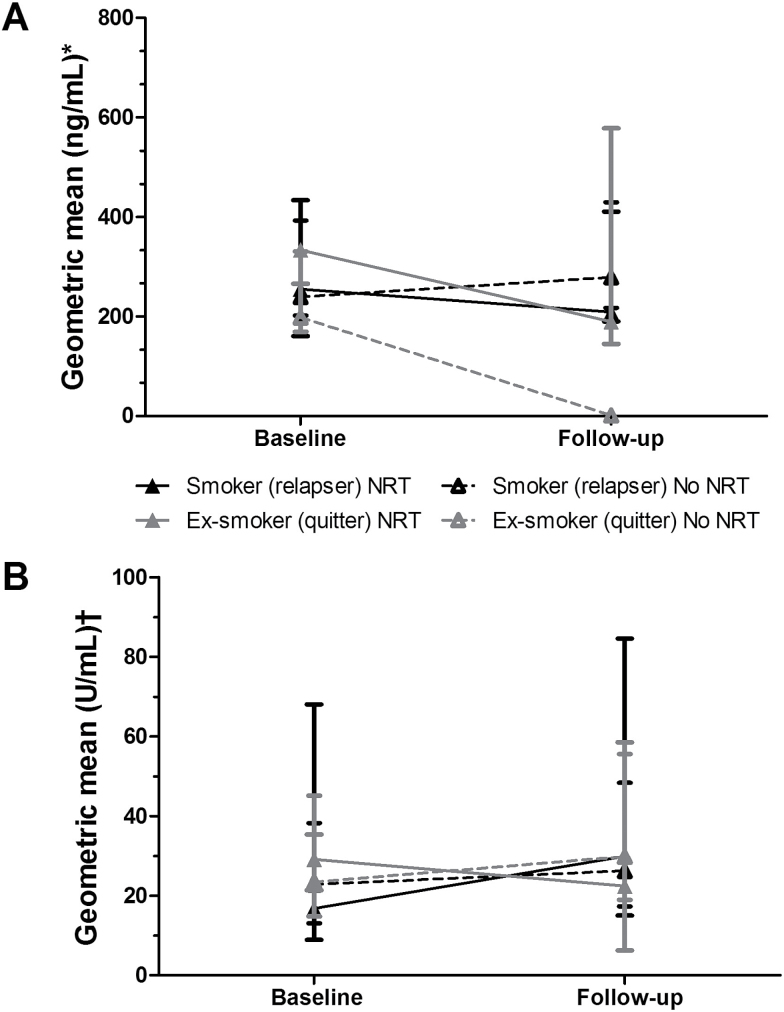
Change in (A) cotinine levels (*N* = 232) and (B) alpha-amylase activity (*N* = 166) from baseline to follow-up as a function of nicotine replacement therapy (NRT) use and smoking status at follow-up. Data not available from *N* participants due to insufficient samples or contamination: *26 cases; †92 cases; Error bars are interquartile range.

In addition to the cross-sectional analyses for baseline and follow-up data reported above, we also examined within-person changes from baseline to follow-up in longitudinal analysis (please note that this group is slightly smaller as not all participants who provided both baseline and follow-up saliva samples had provided either two samples at each time point or samples that were viable). As shown in Figure 2A, cotinine levels significantly reduced from baseline to follow-up only in ex-smokers not using NRT at follow-up (Standardized *Z* = −9.9, *p* < .001) and not in other groups. Adjusted analysis confirmed the significant NRT use by smoking status interaction for changes in cotinine levels (Wald χ^2^ (1) = 13.0, *p* < .001) and also showed that greater baseline age (Wald χ^2^ (1) = 4.3, *p* = .037) and dependence (Wald χ^2^ (1) = 44.8, *p* < .001) were associated with an increase in cotinine levels.

While unadjusted analysis indicated that there was an increase in alpha-amylase activity from baseline to follow-up in ex-smokers not using NRT at follow-up (Standardized *Z* = 3.0, *p* = .003) and not in other groups (Figure 2B), this was not confirmed in adjusted analysis. Neither the NRT use by smoking status interaction for changes in alpha-amylase levels (Wald χ^2^ (1) = 2.1, *p* = .147) nor main effects for NRT use (Wald χ^2^ (1) = 0.9, *p* = .352) or smoking status (Wald χ^2^ (1) = 0.8, *p* = .373) were significant. However, greater baseline age (Wald χ^2^ (1) = 4.4, *p* < .036), dependence (Wald χ^2^ (1) = 6.0, *p* = .014) and reporting any medical condition at baseline (Wald χ^2^ (1) = 5.8, *p* = .016) were independently associated with an increase in alpha-amylase activity.

## Discussion

Extended use of NRT among SSS clients was relatively prevalent, with over one in five who achieve short-term abstinence continuing to use it beyond the standard treatment length of 3 months, but continued long-term use of NRT by those who achieve long-term abstinence at one year is less common at just below 10%. Nonetheless, given that 1-year usage rates were estimated at around 5% among ex-smokers who attend SSS in 2002,^35^ this suggests that recent policy and licensing changes in favor of harm reduction^9,10,12^ may have had some impact on long-term NRT use among services users. This contrasts with a lack of change in NRT usage pattern observed in the general population following an earlier relaxation of NRT licensing in 2005.^36^ However, the low 4% prevalence of concurrent long-term use of NRT among SSS clients who had relapsed is similar to figures from the general population suggesting that longer-term NRT use among smokers is rare.^37^ Indeed, concurrent NRT use among smokers, either for temporary abstinence or cutting down, has remained relatively stable since 2002,^38^ with most smokers using NRT for less than 3 months.^37^

Interestingly, despite a steady increase in the prevalence of e-cigarette use among smokers and ex-smokers in the United Kingdom,^39^ the long-term use of e-cigarettes among past SSS clients in this study was surprisingly low at less than 3% compared with estimates of one in five smokers or recent ex-smokers using e-cigarettes in the general population.^40^ However, this may be due to the specificity of the sample selection and the timing of the study, being conducted around the time of increasing awareness of e-cigarettes in the United Kingdom but before use became widespread amongst smokers and recent quitters.^41^

This study provides some rare insights in the exposure to nicotine associated with long-term dual or single use of NRT, as well as its impact on a biological index of stress, alpha-amylase. Clinical trials suggest that permanent replacement of cigarettes with NRT among ex-smokers can result in 40% of baseline levels of nicotine being substituted by nicotine replacement products long-term.^42,43^ Our findings not only confirm substantial substitution of nicotine from cigarettes with nicotine from NRT but, given the lack of changes in ex-smokers using NRT from baseline to follow-up, suggest that virtually all baseline nicotine may be replaced by NRT among long-term ex-smokers. This increase in substitution levels compared with previous work may reflect differences in our sample or changes in the NRT products available. It is unlikely to be the result of other product use as all ex-smokers were carbon monoxide-verified and participants with concurrent use of other nicotine delivery devices, that is, e-cigarettes, were excluded.

Confirming previous research,^5,44^ the concurrent use of NRT among smokers did not appear to increase their nicotine intake. These findings are in agreement with the hypothesis that smokers are very adept at titrating nicotine levels, with some nicotine otherwise obtained from cigarettes being replaced by nicotine from NRT.^45^ However, our results indicate this also applies to ex-smokers, which is consistent with a strong genetic component in nicotine intake^46^ but at odds with clinical^42^ and general population studies^6^ showing that nicotine substitution from NRT tapers off over time. Behavioral support in SSS includes detailed instructions on the correct use of NRT^47^ which is not available in other settings and may explain the differential in both NRT effectiveness and associated nicotine intake when used with and without behavioral support.

Although it is unlikely that a substantially increased nicotine intake from NRT would be harmful,^48,49^ it clearly is a concern for some people and a potential barrier to effective use of nicotine products.^16^ Our results not only suggest that dual use with NRT does not increased nicotine intake compared with continued smoking, they also indicate that use of NRT (either with or without concurrent smoking) is not associated with an increase in a biomarker of stress response, alpha-amylase, used as a proxy here to signal potential harm. Given observed reductions in stress levels in smokers following cessation,^50^ it was surprising not to see any reductions of alpha-amylase levels in quitters. However, it should be noted that tobacco smoke has been shown to acutely inhibit alpha amylase activity,^33^ which means that the benefit of smoking cessation may have been masked by the impact of baseline smoking. Moreover, spot sampling may not be reliable enough to pick up true long-term changes. While there was an expected association of increased biological stress with older age and having a medical condition, the association of increased alpha-amylase activity with greater baseline nicotine dependence was not predicted and deserves further investigation as it suggests that the stress response is dependence-mediated (rather than nicotine-mediated). Altogether, these findings are consistent with the view that long-term NRT use is safe and not associated with increased health risks, certainly compared with continued smoking.^21^

This study has a number of limitations. Despite an initial large sample size, drop out across the study was inevitably substantial, resulting in relatively few clients with complete baseline and follow-up data on biomarkers. In addition, the baseline sample differed from the sample followed up. However, differences were relatively modest, and prevalence data were weighted to account for differential drop out. As clients self-selected into groups rather than being experimentally assigned, we cannot exclude potential reverse causation, for example, particular individuals who happen to have a high sensitivity to nicotine intake may use NRT for longer. Moreover, we were only able to assess current NRT use but not frequency of NRT use at follow-up which means that it is difficult to ascertain how comparable NRT use was across relapsers and quitters. However, this study reflects real-world use of NRT and the longitudinal within-group design reduced confounding by allowing participants to be their own control. Lastly, different methodologies were used to collect follow-up saliva samples which may have impacted results. However, the same clear instructions were provided to participants and researchers for postal and face-to-face collection, respectively. All assessments were carried out with established, ecologically valid measures and smoking status verified, but further research would benefit from measuring a wider array of biomarkers of smoking-related harm, including different biomarkers of chronic stress such as cortisol.

In conclusion, among former SSS clients long-term NRT use by ex-smokers is relatively rare but more common than use by smokers. Furthermore, long-term use seems to have increased since the introduction of harm reduction guidance in the United Kingdom. Long-term use of NRT does not appear to have a detrimental effect on chronic stress response among smokers or ex-smokers and does not increase overall nicotine intake in smokers but is associated with continued nicotine intake in ex-smokers, comparable to when they were smoking.

## Funding

The ELONS study was funded by the NIHR HTA programme (09/161/01). This work received additional support from a grant by the former UK Centre for Tobacco Control Studies (UKCTS). Funding from the British Heart Foundation, Cancer Research UK, Economic and Social Research Council, Medical Research Council and the National Institute for Health Research under the auspices of the UK Clinical Research Collaboration is gratefully acknowledged (RES-590-28-0004). All authors are also members of the UK Centre for Tobacco and Alcohol Studies (UKCTAS), funded under the auspices of the above UK Clinical Research Collaboration (MR/K023195/1).

## Declaration of Interests

*LS has received an honorarium for a talk, an unrestricted research grant and travel expenses to attend meetings and workshops from Pfizer, a pharmaceutical company that makes smoking cessation products, and has acted as paid reviewer for grant awarding bodies and as a paid consultant for health care companies. The other authors have no conflicts of interest to declare.*
